# Reciprocal interference between the NRF2 and LPS signaling pathways on the immune‐metabolic phenotype of peritoneal macrophages

**DOI:** 10.1002/prp2.638

**Published:** 2020-08-13

**Authors:** Federica Mornata, Giovanna Pepe, Chiara Sfogliarini, Electra Brunialti, Gianenrico Rovati, Massimo Locati, Adriana Maggi, Elisabetta Vegeto

**Affiliations:** ^1^ Center of Excellence on Neurodegenerative Diseases University of Milan Milan Italy; ^2^ Department of Pharmaceutical Sciences University of Milan Milan Italy; ^3^ Department of Health Sciences University of Milan Milan Italy; ^4^ Department of Medical Biotechnologies and Translational Medicine University of Milan Milan Italy; ^5^ Humanitas Clinical and Research Center‐ IRCCS Rozzano Italy

**Keywords:** immunometabolism, inflammation, NFκB, NRF2, resident macrophages

## Abstract

The metabolic and immune adaptation to extracellular signals allows macrophages to carry out specialized functions involved in immune protection and tissue homeostasis. Nuclear factor erythroid 2‐related factor 2 (NRF2) is a transcription factor that coordinates cell redox and metabolic responses to stressors. However, the individual and concomitant activation of NRF2 and inflammatory pathways have been poorly investigated in isolated macrophages. We here took advantage of reporter mice for the transcriptional activities of NRF2 and nuclear factor‐kB (NFκB), a key transcription factor in inflammation, and observe a persisting reciprocal interference in the response of peritoneal macrophages to the respective activators, tert‐Butylhydroquinone (tBHQ) and lipopolysaccharide (LPS). When analyzed separately by gene expression studies, these pathways trigger macrophage‐specific metabolic and proliferative target genes that are associated with tBHQ‐induced pentose phosphate pathway (PPP) with no proliferative response, and with opposite effects observed with LPS. Importantly, the simultaneous administration of tBHQ + LPS alters the effects of each individual pathway in a target gene‐specific manner. In fact, this co‐treatment potentiates the effects of tBHQ on the antioxidant enzyme, HMOX1, and the antibacterial enzyme, IRG1, respectively; moreover, the combined treatment reduces tBHQ activity on the glycolytic enzymes, TALDO1 and TKT, and decreases LPS effects on the metabolic enzyme IDH1, the proliferation‐related proteins KI67 and PPAT, and the inflammatory cytokines IL‐1β, IL‐6, and TNFα. Altogether, our results show that the activation of NRF2 redirects the metabolic, immune, and proliferative response of peritoneal macrophages to inflammatory signals, with relevant consequences for the pharmacological treatment of diseases that are associated with unopposed inflammatory responses.

## Graphical abstract

**NRF2 and LPS signaling pathways in peritoneal macrophages. **

Together with the anti‐oxidant response, the administration of the NRF2‐inducer, tBHQ, regulates macrophage energy request and the inflammatory phenotype, by increasing energy fuelling through the PPP, reducing the TCA and inhibiting the production of inflammatory mediators. Treatment with LPS stimulates the cell metabolic adaptation, to provide the energy support for bactericidal, inflammatory and prolfierative activities through the alteration of the PPP, truncation of the TCA and expression of proliferative and inflammatory effectors. The concomitant activation of the NRF2 and LPS signalling pathways potentiates the antioxidant and bactericidal responses and reduces the metabolic, phenotypic and proliferative effects activated by each individual pathway.

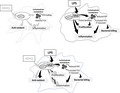

## 1 INTRODUCTION

Tissue‐resident macrophages, such as those populating the peritoneal cavity or brain, derive from progenitor cells that self‐renew and differentiate locally under the influence of microenvironmental cues.^1^, ^2^ These immune cells are extremely reactive and, in response to a broad variety of physiological and pathological stimuli, activate a spectrum of different transcriptional programs and functional responses, simplistically included within two extreme phenotypes defined as “classical”, or M1, and “alternative”, or M2, activation.^3^ Binding of macrophages to pathogens induces a series of antibacterial responses through the activation of transcription factors, among which nuclear factor‐kB (NFκB) plays a central role, and the robust production of inflammatory cytokines, such as IL‐1β, IL‐6, and TNFα. On the other hand, Th2 cytokines induce the expression of proteins, such as arginase 1 (ARG1), that trigger the anti‐inflammatory and tissue repairing activities of M2 macrophages.^4^

In order to meet the energy demand required for functional specialization, M1 macrophages are characterized by a metabolic shift toward aerobic glycolysis, carbon flux through the pentose phosphate pathway (PPP), and fatty acid synthesis, with truncated TCA cycle, which allows to rapidly produce energy for bactericidal functions and self‐renewal. Hence, along with inflammatory mediators, also robust changes in the expression of metabolic enzymes accompany the M1 polarization of macrophages and even sustain their immune function. In fact, the mitochondrial metabolite itaconate is endowed with bactericidal activity and derives from the activity of the enzyme IRG1 whose expression is highly induced in inflammatory macrophages.^5^, ^6^, ^7^, ^8^ Thus, elucidating the interconnections between immune and metabolic responses in macrophages might help in understanding the physiology of cell adaptation and the pathological consequences of unopposed or elusive inflammatory responses.^9^

Nuclear factor erythroid 2‐related factor 2 (NRF2) is a transcription factor deeply implicated in cell adaptation to stressors. Under unstimulated conditions, binding to the cytoplasmic protein KEAP1 leads to NRF2 degradation. Exposure to oxidative or electrophilic compounds, such as tert‐Butylhydroquinone (tBHQ), disrupts KEAP1‐NRF2 binding and allows NRF2 to migrate to the nucleus and bind antioxidant responsive elements (AREs) within target genes, following heterodimerization with MAF proteins.^10^ The resulting transcriptional program allows detoxification from the initial chemical stressor and the activation of cytoprotective antioxidative reactions. More recent studies, using animal models of dysregulated inflammation which also carry genetic manipulations of the KEAP1‐NRF2 complex, demonstrated that NRF2 is also necessary for mounting an appropriate innate immune response, as its activity has been associated with a reduced production of pro‐inflammatory cytokines during inflammation.^11^, ^12^, ^13^, ^14^, ^15^ In general, the metabolic shift that accompanies cell adaption to stressors has been associated with NRF2‐mediated generation of anabolic precursors and NADPH.^16^, ^17^ However, the specific metabolic response of macrophages and their adaptation to the simultaneous activation of NRF2 and inflammatory pathways needs further investigation, as mostly approached with experimental systems, such as in vitro differentiated cells or cell lines, that profoundly differ from resident macrophages, particularly in cell phenotype and immunometabolism. Instead, it is important to deciphering the complexity of macrophage immune‐metabolic network under multiple activatory stimuli and through the use of a more reliable experimental model of tissue‐resident macrophages.

The aim of the present study is thus to use primary cultures of peritoneal macrophages and evaluate their immune and metabolic responses to the NRF2‐activator, tBHQ, and the bacterial wall component, lipopolysaccharide (LPS). Exploiting the availability of NRF2 and NFκB reporter mice, we here show that the concomitant administration of tBHQ and LPS to macrophages modifies the antioxidant, metabolic, inflammatory, and proliferative responses induced by each individual signal and suggest that NRF2 is able to redirect macrophage immunometabolism during infections or inflammatory conditions.

## 2 METHODS

### 2.1 Animals and treatment

C57BL/6 female mice of 4 months of age were supplied by Charles River Laboratories (Calco, Italy) and used for gene expression analyses. Animals were allowed to food and water access ad libitum and kept in temperature‐controlled facilities on a 12‐hour light and dark cycle. Animals were housed in the animal care facility of the Department of Pharmacological and Biomolecular Sciences at the University of Milan. Animal investigation has been conducted in accordance with the ethical standards and according to the Declaration of Helsinki and to the Guide for the Care and Use of Laboratory Animals, as adopted and promulgated by the US National Institute of Health, and in accordance with the European Guidelines for Animal Care and Use of Experimental Animals. Animals were sacrificed by a lethal ketamine/xylazine solution (150 and 12 mg/kg, respectively). Generation of NFκB‐Luc and ARE‐luc2 animals has already been described^18^, ^19^; female mice were used at 4 months of age. All animal experiments were approved by the Italian Ministry of Research and University and controlled by an academic panel of experts.

### 2.2 Primary cultures of peritoneal macrophages

Peritoneal cells were recovered by peritoneal lavage as previously described.^20^ Briefly, 5 mL of prechilled 0.9% NaCl was injected into the peritoneal cavity using a 21 G needle, and cell suspension was recovered and centrifuged; following incubation with ACK solution (0.15‐mol/L NH_4_Cl, 1‐mmol/L KHCO_3_, and 0.1‐mmol/L EDTA; pH 7.3) for 5 minutes at 4°C, cells were seeded at the concentration of 1 × 10^6^ cells/mL in RPMI (Life Technology‐Invitrogen) supplemented with 10% endotoxin‐free FBS, 1% penicillin/streptomycin, and 1% Na‐pyruvate. After 45 minutes and several washes in PBS, the medium was replaced with RPMI w/o phenol red supplemented with 10% dextran‐coated charcoal (DCC)‐FBS (RPMI + 10% DCC). On the next day, cells were treated with vehicle (DMSO 20% in H_2_O), tBHQ (100 µmol/L), LPS (1 µg/mL), or the combination of the two stimuli for 3, 6, or 16 h, as specified in each experiment. Cell suspension was centrifuged at 1200× *g*, cell pellets were re‐suspended in TRIzol reagent (Life Technology‐Invitrogen), and stored at −80°C for RNA, while supernatant was stored for ELISA analyses. tBHQ induces a strong antioxidant response through mitochondrial oxidative stress and NRF2 activation,^21^ without inducing cell toxicity in our experimental conditions (data not shown).

### 2.3 RNA preparation and expression analyses

Total RNA was purified using Direct‐zol RNA Miniprep (Zymo Research), according to the manufacturer's instructions, including a step with deoxyribonuclease incubation. For real‐time PCR, 200‐ng RNA was used for cDNA preparation using 8 U/μL of Moloney murine leukemia virus reverse transcriptase (Promega) in a final volume of 25 μL. The reaction was performed at 37°C for 1 hour, and the enzyme was inactivated at 75°C for 5 minutes. Control reactions without the addition of the reverse transcription enzyme were performed (data not shown). A 1:4 cDNA dilution was amplified using GoTaq^®^ qPCR Master Mix technology (Promega) according to the manufacturer's protocol. The PCR was carried out in triplicate on a 96‐well plate using QuantStudio^®^ 3 real‐time PCR system (Applied Biosystems) with the following thermal profile: 2 minutes at 95°C; 40 cycles, 15 seconds at 95°C, and 1 minutes at 60°C. Primer sequences are reported in Table S1. Data were analyzed using the 2^−ΔΔCt^ method and normalized using 36b4 as housekeeping gene.

### 2.4 Luciferase enzymatic assay

Cells were lysed with Luciferase Cell Culture Lysis Reagent (Promega) and the luciferase assay was carried out in luciferase assay buffer (470‐μm luciferin, 20‐mm Tricine, 0.1‐mm EDTA, 1.07‐mm (MgCO3)4·Mg(OH)2 × 5H2O; 2.67‐mm MgSO4 × 7H2O in H2O, pH 7.8, with 33.3‐mm DTT and 530‐μm ATP), using 20 μL of cell lysate and 100 μL of luciferase assay buffer. Luminescence emission was measured with a Veritas luminometer (Promega). Protein concentration was determined by the Bradford assay and used to normalize luciferase units/μg protein, obtaining the relative luminescence units (RLU).

### 2.5 ELISA assay

Culture medium from peritoneal macrophages treated with different combinations of tBHQ and LPS was assayed for IL‐1β, IL‐6, and TNFα protein levels by enzyme immunoassay (murine ELISA kits from Bio‐Techne). Dilutions of 1:10 were made for IL‐6 and TNFα assays.

### 2.6 Statistical analyses

All data are presented as mean ± SEM of three observations. Unless otherwise indicated, results were analyzed by one‐way ANOVA followed by Bonferroni multiple comparison test. A statistical level of significance of *P* < .05 was accepted. Statistical analysis was performed using GraphPad Prism software, version 8.

## 3 RESULTS

### 3.1 Reciprocal interference between NRF2 and NFκB transcriptional activities in macrophages

**FIGURE 1:**
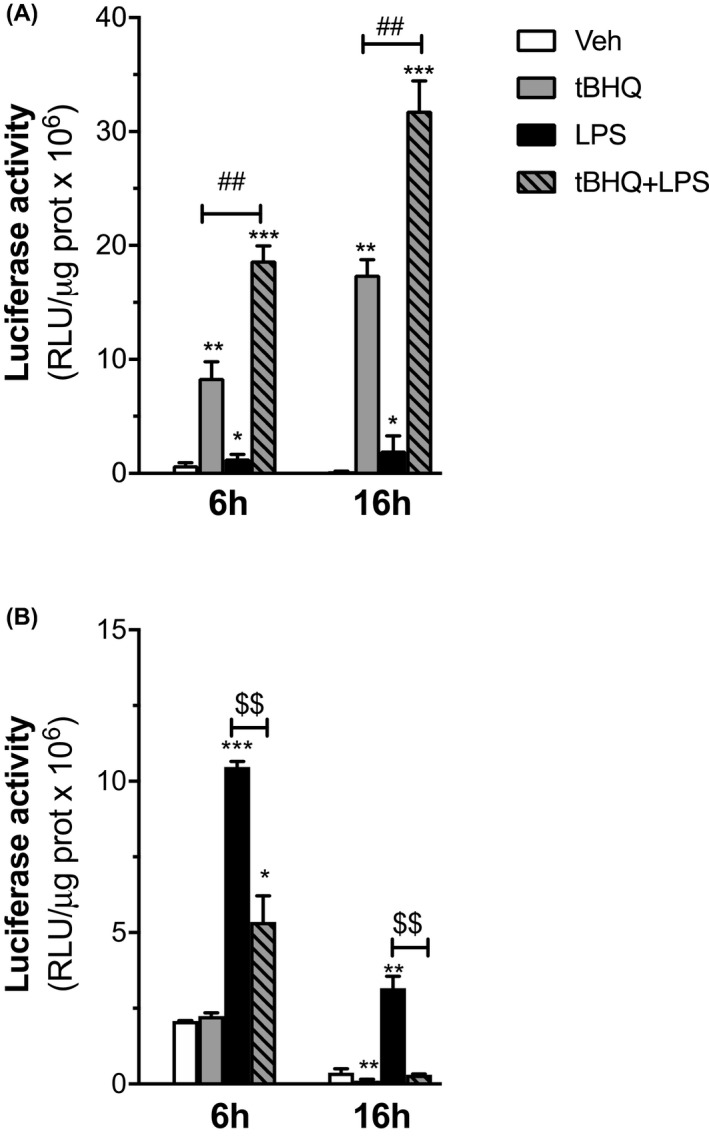
Regulation of luciferase reporter activity by oxidative and inflammatory stimuli in peritoneal macrophages from ARE‐*luc2* and NFκB‐*luc2* mice. Peritoneal macrophages isolated from ARE‐*luc2* (A) and NFκB*‐luc2* (B) reporter animals were used to assay NRF2 and NFκB transcriptional activity. Cells were treated with vehicle (veh, open boxes), tBHQ (grey boxes), LPS (filled boxes), or the combination of the two stimuli (dashed grey boxes) and luminescence measured after 6 or 16 h, as indicated. Luciferase activity is represented as relative luciferase units (RLU) per μg protein and expressed in relation with 6‐h veh‐treated samples, which was given the arbitrary value of 1. Data are presented as mean values ± SEM (n = 3) of a single experiment representative of at least two other independent experiments. Results were analyzed by one‐way ANOVA followed by Bonferroni's multiple comparisons test. (*df* = 11; A) 6 h: *F* = 351.2, 16 h: *F* = 405.3; B) 6 h: *F* = 355.6, 16 h: *F* = 233.9). (*) *P* < .05, (**) *P* < .01, (***) *P* < .001 vs 6 h veh; (*^##^*) *P* < .001 vs tBHQ; (^$$^) *P* < .001 vs LPS

Although cell metabolism and immune functions are strictly interconnected in macrophage physiology, the effects of the concomitant activation of inflammatory and NRF2‐activating signals in the immunometabolic adaptation of resident macrophages are still poorly defined. We first took advantage of the ARE‐luc2 reporter mice, a transgenic strain engineered to express enzyme luciferase under the control of AREs‐containing promoter,^18^ to readily obtain biological evidence of the influence of inflammatory signals on NRF2 activity. Primary cultures of peritoneal macrophages were obtained from ARE‐luc2 mice, treated with tBHQ and LPS, either alone or in combination, and protein extracts were prepared after 6 and 16 hours to measure bioluminescence emission. As expected, treatment with tBHQ results in the increase of luciferase activity that is proportion with the time of incubation, reflecting the induction of NRF2‐driven transcription (see Figure 1A). Also, LPS induces similar although much weaker effects as compared with tBHQ, supporting the notion that inflammatory signals induce NRF2 transcriptional activity in macrophages. Importantly, the tBHQ + LPS treatment potentiates both the short‐ and long‐term effects of tBHQ alone on NRF2 transcriptional activity, suggesting that inflammatory conditions are able to influence the pharmacologically induced activity of NRF2 in macrophages.

We then asked whether the pharmacological activation of NRF2 causes the metabolic and immune reprograming of M1 macrophages. To this aim, we used the NFκB‐luc2 reporter mice, engineered to express the luciferase enzyme under the control of a promoter containing NFκB‐responsive elements.^19^ NFκB‐luc2 peritoneal macrophages were treated as reported above. As expected, LPS induces luciferase activity in a time‐dependent manner, with 10‐ and 3‐fold inductions observed after 6 and 16 hours, respectively (see Figure 1B). Both short and prolonged NFκB‐mediated transcriptional effects are reduced with the tBHQ + LPS treatment; interestingly, tBHQ alone also provides inhibitory effects at the later time point analyzed. Thus, these results suggest that the pharmacological activation of NRF2 modifies the inflammatory response of macrophages to LPS by triggering significant and persisting inhibitory effects on NFκB transcriptional activity.

### 3.2 Validation of NRF2 target genes in macrophages

**FIGURE 2:**
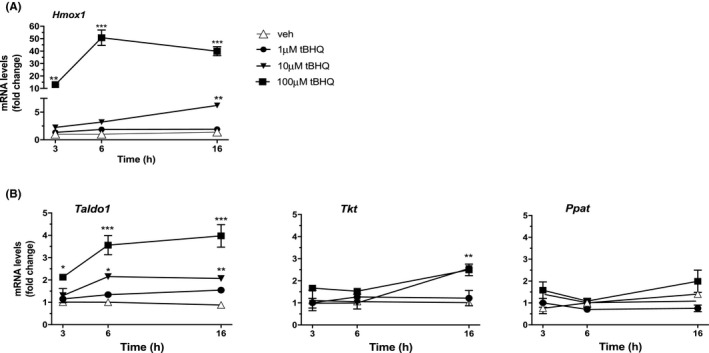
Time‐ and dose‐dependent effect of NRF2 activation on NRF2 target genes in peritoneal macrophages. The expression of candidate NRF2 target genes was measured in peritoneal macrophages treated with vehicle (open triangles) or increasing concentrations of tBHQ (1 µmol/L, filled circles; 10 µmol/L, filled triangles; 100 µmol/L, filled squares) for 3, 6, and 16 h, as indicated. Real‐time PCR was used to analyze the mRNA levels coding for (A) HMOX1 and (B) TALDO1, TKT, and PPAT. Data sets for each gene were calculated using the 2^−ΔΔCt^ method and expressed in relation to 3 h veh samples. Data are presented as mean values ± SEM (n = 3) of a single experiment representative of at least two other independent experiments. Results were analyzed by two‐way ANOVA followed by Dunnett's multiple comparisons test (HMOX1, *F*(3,24) = 400.8; TALDO1, *F*(3,24) = 122.9; TKT, *F*(3,24) = 18.56; PPAT, *F*(3,24) = 14.74). (*) *P* < .05; (**) *P* < .01, (***) *P* < .001 vs veh

By coupling an antioxidant activity with a shift in energetic metabolism, the NRF2‐mediated response is a key system in cell protection against oxidative stress. Diverse NRF2 target genes have been identified, depending on the cell type analyzed and specific function required by NRF2 activation. In order to study the endogenous transcriptional activity of NRF2 in peritoneal macrophages, we first performed dose‐ and time‐dependent experiments with tBHQ and assessed the mRNA abundance of candidate target genes. As shown in Figure 2A, the mRNA levels coding the antioxidant protein HMOX1 are readily increased by high concentrations of tBHQ with stronger effects detected at later time points; also *Nqo1* mRNA levels are induced in a time‐ and dose‐dependent manner, although with a lower efficacy (see Figure S1). In parallel, we analyzed the expression of NRF2 target metabolic enzymes, namely transaldolase‐1 (TALDO1) and transketolase (TKT), that sustain the PPP associated with macrophage polarization, as well as phosphoribosyl pyrophosphate amidotransferase (PPAT), a rate‐limiting enzyme in purine biosynthesis which has been reported to be increased by NRF2 activity in proliferating cancer cells.^11^, ^22^ Figure 2B shows that *Taldo1* mRNA levels increase in a dose‐ and time‐dependent manner following NRF2 chemical activation, while later time points and higher concentrations of tBHQ are necessary to increase *Tkt* expression. Unexpectedly, the mRNA levels encoding PPAT are not modified by tBHQ at any concentration used.

Altogether, these experiments provide the dynamics of redox and metabolic gene expression that is induced by the pharmacological activation of NRF2 in peritoneal macrophages.

### 3.3 Modulation of metabolic, cell cycle, and inflammation‐related genes by tBHQ and LPS in macrophages

**FIGURE 3:**
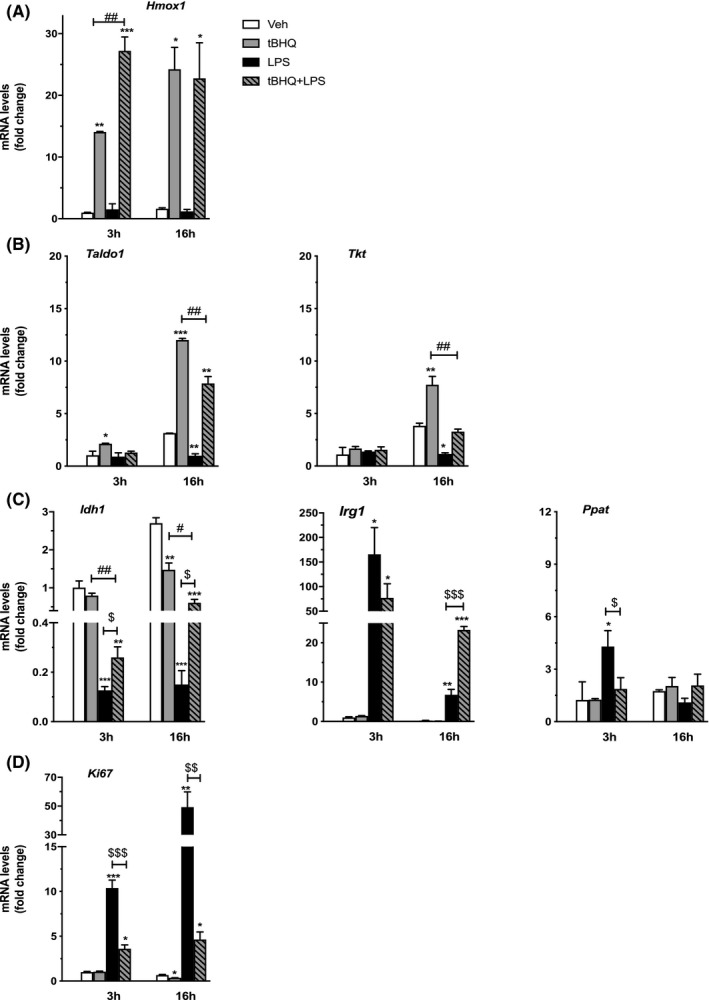
Target gene expression following the concomitant activation of NRF2 and LPS‐activating signals in peritoneal macrophages. The expression of NRF2 and LPS target genes was evaluated in peritoneal macrophages treated with vehicle (veh, white bars), tBHQ (grey bars), LPS (black bars), and the combination of the two stimuli (dashed grey bars) for 3 or 16 h, as indicated. Real‐time PCR was used to analyze the mRNA levels coding (A) HMOX1 (B, C) TALDO1, TKT IDH1, IRG1, and PPAT, and (D) KI67. Data sets for each gene were calculated using the 2^−ΔΔCt^ method and expressed in relation to 3 h veh samples. Data are presented as mean values ± SEM (n = 3) of a single experiment representative of at least two other independent experiments. Results were analyzed by one‐way ANOVA followed by Bonferroni's multiple comparisons test. (*df* = 11; HMOX1, 3 h: *F* = 0.7, 16 h = 1.074; TALDO1, 3 h: *F* = 8.255, 16 h: *F* = 869.2; TKT, 3 h: *F* = 1.114, 16 h: *F* = 188.7; IDH1, 3 h: *F* = 93.44, 16 h: *F* = 353.1; IRG1, 3 h: *F* = 21.25, 16 h: *F* = 803.1; PPAT, 3 h: *F* = 12.73, 16 h: *F* = 5.013; KI67 3 h: *F* = 374.2, 16 h: *F* = 100.6). (*) *P* < .05, (**) *P* < .01, (***) *P* < .001 vs 6 h veh; (^###^) *P < *.001 vs tBHQ; (^$$$^) *P* < .001 vs LPS

Metabolic adaptation and immune activation are interlinked and concomitant events in macrophage immune response. The results of Figure 1 lead us to predict that inflammatory conditions and NRF2 activation reprogram the transcriptional effects induced by each signaling pathway, causing a reciprocal interference with macrophage immunometabolism. Separated or combined treatments with tBHQ and LPS were used in time‐course experiments to assess NRF2 and LPS target gene expression. In line with the positive effects of LPS on NRF2 activity shown in Figure 1A, we observed that the increase in *Hmox1* mRNA induced by short‐term tBHQ treatment potentiates the inflammatory signal (see Figure 3A). On the contrary, the combined tBHQ + LPS treatment causes a reduction in the effects of tBHQ alone on the expression of the NRF2 target metabolic enzymes TALDO1 and TKT (see Figure 3B). Unexpectedly, we also observed that treatment with LPS alone significantly reduces *Taldo1* and *Tkt* mRNA levels, while it increases the expression of PPAT, an enzyme involved in de novo purine biosynthesis, suggesting that LPS may reduce energy fueling through the PPP, as already reported,^23^ and support cell proliferation, which still needs biological confirmation. Thus, these results show that LPS regulates gene expression in a peritoneal macrophage‐specific manner and that it is able to modify the transcriptional effects of NRF2 associated with cell redox and metabolic adaptation.

To analyze the NRF2‐mediated interference with the metabolic effects of LPS, we took advantage of the fact that the metabolic signature of M1 macrophages is characterized, among others, by a truncated TCA cycle.^24^ We thus assayed the expression of isocitrate dehydrogenase (IDH1) and immune‐responsive gene 1 (IRG1), two key enzymes in macrophage TCA cycle that display opposite activities on citrate metabolism.^7^, ^25^ According to published data,^9^ LPS downregulates the mRNA levels of *Idh1* and strongly upregulates those coding *Irg1* in peritoneal macrophages, as shown in Figure 3C. More importantly, these effects are significantly different following the combined treatment with LPS + tBHQ. In fact, this association reduces the inhibitory effects of LPS on *Idh1* and potentiates its long‐term effects on *Irg1* mRNA levels.

**FIGURE 4:**
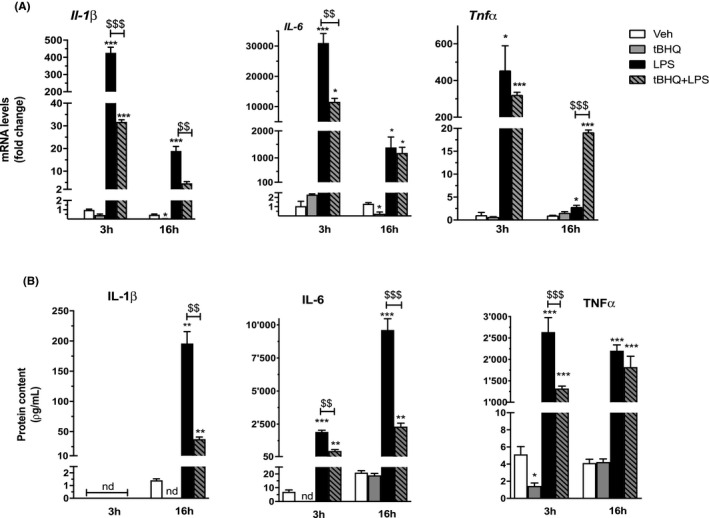
NRF2‐mediated interference with inflammatory gene expression induced by LPS in peritoneal macrophages. The expression of inflammatory genes was evaluated in peritoneal macrophages treated with vehicle (veh, white bars), tBHQ (grey bars), LPS (black bars), and the combination of the two stimuli (dashed grey bars) for 3 or 16 h, as indicated. A, Real‐time PCR was used to analyze the mRNA levels coding IL‐1β, IL‐6, and TNFα. Data sets for each gene were calculated using the 2^−ΔΔCt^ method and expressed in relation to 3 h veh samples. (B) IL‐1β, IL‐6, and TNFα proteins were analyzed by ELISA immunoassay in the culture medium of peritoneal macrophages treated as in (A). Data are presented as mean values ± SEM (n = 3) of a single experiment representative of at least two other independent experiments. Results were analyzed by one‐way ANOVA followed by Bonferroni's multiple comparisons test (in A) *df* = 11; IL‐1β, 3 h: *F* = 482, 16 h: *F* = 203.7; IL‐6 3 h: *F* = 332.3, 16 h: *F* = 48.32; TNFα 3 h: *F* = 58.70, 16 h: *F* = 3367; in (B), IL‐1β, *df* = 8, *F* = 244.9; IL‐6, 3 h: *df* = 8, *F* = 501.4, 16 h: *df* = 11, *F* = 435.7; TNFα *df* = 11, 3 h: *F* = 231.8, 16 h: *F* = 311.1). (*) *P* < .05; (**) *P* < .01; (***) *P* < .001 vs veh; (^$$^) *P* < .01; (^$$$^) *P* < .001 vs LPS

Since metabolic adaptation to LPS is supposed to correlate with cell proliferation, we assessed the expression of KI67, a marker of proliferating cells. The results are reported in Figure 3D and show that LPS induces an immediate and persistent increase in *Ki67* expression and that the co‐administration of tBHQ significantly reduces this effect, in agreement with what is shown in Figure 4C on the expression of PPAT, the enzyme involved in de novo purine biosynthesis. These results suggest that inflammatory stimuli sustain the proliferation of peritoneal macrophages, a response that is significantly reduced by the simultaneous activation of NRF2.

Since metabolic adaptation is strongly connected to immune activation, we extended our analyses to inflammatory gene expression. As expected, treatment of peritoneal macrophages with LPS increases the mRNA levels coding IL‐1β, IL‐6, and TNFα. Interestingly, we observed a significant reduction of *Il‐1β* and *Il‐6* mRNA levels following tBHQ + LPS as compared to LPS alone, with a more persistent inhibition *of IL‐1β*. We also analyzed TNFα, since its expression is differentially regulated by inflammatory stimuli.^26^ Indeed, we observed that the combined tBHQ + LPS treatment only slightly reduces the early effect of LPS, without reaching statistical significance, on *TNFα* mRNA levels; conversely, it increases the response to the endotoxin at the later time point (see Figure 4A). Notably, when added alone, tBHQ also induces a delayed reduction of *Il‐1β* and *Il‐6* mRNA levels, as compared to controls. In order to obtain a biological evidence for the immune regulatory effects of NRF2 on macrophage immune activation, we assessed the cytokine protein levels that were present in the culture medium of macrophages treated as above. The results reported in Figure 4B confirm the data on gene expression and show that the combined treatment with tBHQ + LPS significantly reduces the levels of IL‐1β and IL‐6. Interestingly, also TNFα levels are reduced at the early time point of tBHQ and endotoxin treatment, indicating an early transcriptional interference on this gene, which is counteracted at later time points only at the mRNA level. In addition, we observed a consistent reduction, beneath measurable levels, of IL‐1β, IL‐6, and TNFα when tBHQ was added alone to the cells. Thus, NRF2 activation during the inflammatory response induces anti‐inflammatory effects through gene‐specific dynamic effects.

Altogether, these results show that the NRF2 and LPS pathways, when analyzed separately, induce a macrophage‐specific subset of target genes, and that the inflammatory response of resident macrophages can be regulated by the pharmacological activation of NRF2 by reprograming the expression of metabolic, proliferative, and inflammatory genes.

## 4 DISCUSSION

NRF2 is a key transcriptional regulator of detoxification and redox balance that protects cells against chemical stressors. Recently, NRF2 became an attractive target for immune‐regulatory therapies, as a negative role for NRF2 activation on inflammatory responses was demonstrated using in vivo LPS‐mediated animal models or in vitro differentiated macrophages, also bearing *Keap1* and *Nrf2* gene deletions.^14^, ^27^ Our results extend this knowledge to peritoneal macrophages and show that the pharmacological activation of NRF2 interferes with the macrophage response to LPS, not only by reducing inflammatory gene expression but also by altering the expression of metabolic enzymes involved in cell phenotypic activation.

Initially, the anti‐inflammatory activity of NRF2 has been ascribed to the upregulation of antioxidant enzymes that are able to eliminate ROS; more recently, NRF2 has been shown to inhibit the activity of the inflammatory transcription factor, NFκB.^12^ The use of reporter mice in the present study allowed us to confirm this hypothesis, as we observed a persistent reduction of NFκB transcriptional activity in response to NRF2 activation. Moreover, we here show that the inhibitory effects of the combined tBHQ + LPS treatment, as compared with those induced by the two individual signals, occur on both antioxidant and inflammatory genes shortly after their administration to macrophages (see Figures 3A and 4), suggesting that the downstream biochemical processes activated by this treatment proceed in parallel and, probably, independently from each other. The molecular mechanisms underlying the interference between NRF2 and LPS‐induced inflammatory mediators, such as NFκB, are still not fully elucidated. Previous evidence showed that NRF2 is able to physically interact with Pol II and reduce its binding to inflammatory gene promoters, or with anti‐inflammatory transcription factors, such as the estrogen and peroxisome proliferator‐activated gamma receptors, and regulate their activity on gene expression.^12^, ^28^, ^29^ Further studies are needed to understand the molecular mechanism of NRF2 activity in macrophages.

It has been shown that selected metabolic molecules produced by NRF2 activation in response to inflammatory signals are able to switch macrophages toward an anti‐inflammatory phenotype.^27^ We here further extend this hypothesis and demonstrate that also the expression of metabolic genes regulated by NRF2 or LPS is reciprocally influenced by the co‐occurrence of NRF2 and inflammatory‐activating signals. This leads us to propose that the shift in energy metabolism and consumption allows NRF2 to induce an integrated cellular response that redirects macrophage polarization toward an anti‐inflammatory phenotype. Interestingly, we observed a potentiation of NRF2 transcriptional activity by LPS, as it results from the use of NRF2 reporter macrophages treated with LPS + tBHQ (Figure 1) that is reflected by the increase in antioxidant gene expression. On the other hand, the NRF2 positive effects on metabolic genes are instead reduced by LPS, further supporting a competition mechanism for gene‐specific transcription factors, which also need further elucidations.

Our results demonstrate a novel activity of NRF2 in inflammatory macrophages that provides an antiproliferative response. The underlying mechanism is still not clear; however, the metabolic effects observed following NRF2 chemical activation are consistent with the absence of DNA replication. In fact, we detected an increased expression of glycolytic enzymes involved in the PPP, TKT, and TALDO1, not paralleled by any effect on PPAT, the rate‐limiting enzyme in the de novo biosynthesis of purines and aromatic amino acids (see Figure 2). Although the function of TKT and TALDO1 in the process of macrophage activation is still not clear, these enzymes operate without the need of energy to provide triose‐phosphates that enter back glycolysis and sustain the cell metabolic switch that is necessary for phenotypic activation.^17^ Thus, our data suggest that NRF2 activation in resident macrophages promotes glucose metabolism through the PPP that is not followed by purine synthesis and cell proliferation. These data are apparently in contrast with previous studies which showed a positive involvement of NRF2 in anabolic processes leading to nucleotides and amino acid synthesis. Although the reasons for this discrepancy are presently unknown, we believe that the use of primary cultures of nonelicited peritoneal macrophages in the present study allows to appreciating the reactivity and specialized functions of this subpopulation of resident immune cells, including self‐renewal, highlighting distinctions from macrophage‐like cells derived from in vitro differentiation of monocytes or bone marrow precursor cells, or transformation into immortalized cell lines.

The choice of the experimental system appears also an asset for the study of macrophage immunometabolism that mimics the pathophysiologic condition. In fact, it appears crucial to understand the ability of macrophages to respond simultaneously to diverse stimuli and coordinate their activation with time, similarly to what is occurring in tissues when multiple and simultaneous activation events skew macrophage immunometabolism by altering the molecular mechanisms that initiate and amplify cell polarization.^22^ This recently expanded field in immunology is gaining even more attention since causal connections are emerging between alterations of resident macrophage immunometabolism and pathologic conditions, such as atherosclerosis and neurodegenerative diseases.^30^, ^31^, ^32^

Importantly, the possibility to activate NRF2 by pharmacologic agents during inflammatory conditions may have interesting consequences for pathologies characterized by unrestrained inflammatory responses. In fact, genetic polymorphisms of the *Nrf2* gene promoter region, that result in reduced expression of this transcription factor, are associated with an increased susceptibility to inflammatory diseases in mouse models and humans.^33^, ^34^ Moreover, endogenous proteins, such as GSK3 and PTEN, reduce NRF2 activation and may be involved in providing unopposed or more efficient inflammatory effects through the inability of NRF2 to counteract macrophage pro‐inflammatory activation.^11^, ^35^ On the other hand, activation of NRF2 has been associated with several diseases, including cancer.^16^, ^36^ Considering the detrimental role played by anti‐inflammatory macrophages in cancer development, NRF2‐mediated interference with classic pro‐inflammatory signaling might influence macrophage immunometabolic conversion and favor the development of a tolerant phenotype that concurs in cancer development.

In summary, our study demonstrates that NRF2 activation by chemical agents modifies immune and metabolic gene expression reducing inflammatory and proliferative responses in peritoneal macrophages. These effects were related to the interference with NFκB and consequent effects on gene expression. This study strongly supports the hypothesis that the pharmacological regulation of NRF2 changes macrophage phenotypic activation, suggesting that NRF2 is a useful target for inflammatory‐related diseases.

## CONFLICT OF INTEREST

The authors declare that no conflict of interest exists.

## AUTHORS' CONTRIBUTIONS

F. Mornata and G.Pepe performed the experiments and analyzed the data. C. Sfogliarini and E. Brunialti helped with discussion of the data of reporter animals and cell culture experiments, respectively. G. Rovati performed the statistical analysis. A. Maggi, M. Locati, and E. Vegeto discussed the data and wrote the manuscript. E. Vegeto conceived the study.

## Supporting information

Figure S1

Table S1

## Data Availability

No data can be shared.
