# Supplementary material for: Reciprocal interference between the NRF2 and LPS signaling pathways on the immune‐metabolic phenotype of peritoneal macrophages
Source: Pharmacol Res Perspect. 2020 Aug 13;8(4):e00638. doi: 10.1002/prp2.638 (PMC7426195; doi:10.1002/prp2.638)
Supplement: Supplementary file 1 — Figure S1 [file PRP2-8-e00638-s001.pptx]

## Slide 1
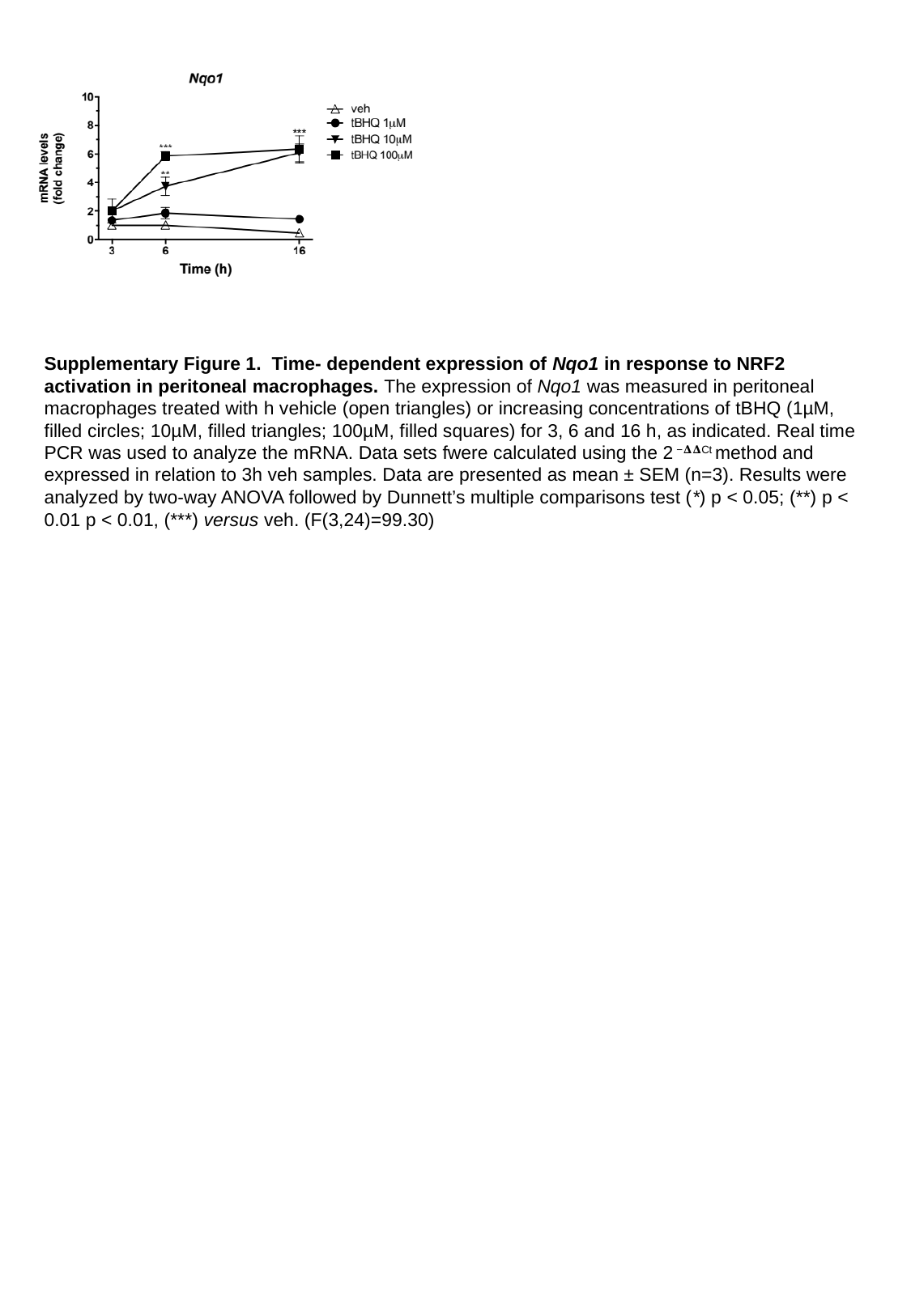

Supplementary Figure 1. Time- dependent expression of Nqo1 in response to NRF2 activation in peritoneal macrophages. The expression of Nqo1 was measured in peritoneal macrophages treated with h vehicle (open triangles) or increasing concentrations of tBHQ (1µM, filled circles; 10µM, filled triangles; 100µM, filled squares) for 3, 6 and 16 h, as indicated. Real time PCR was used to analyze the mRNA. Data sets fwere calculated using the 2 −𝚫𝚫Ct method and expressed in relation to 3h veh samples. Data are presented as mean ± SEM (n=3). Results were analyzed by two-way ANOVA followed by Dunnett’s multiple comparisons test (*) p < 0.05; (**) p < 0.01 p < 0.01, (***) versus veh. (F(3,24)=99.30)
