# Supplementary material for: Reciprocal interference between the NRF2 and LPS signaling pathways on the immune‐metabolic phenotype of peritoneal macrophages
Source: Pharmacol Res Perspect. 2020 Aug 13;8(4):e00638. doi: 10.1002/prp2.638 (PMC7426195; doi:10.1002/prp2.638)
Supplement: Supplementary file 2 — Table S1 [file PRP2-8-e00638-s002.pptx]

## Slide 1
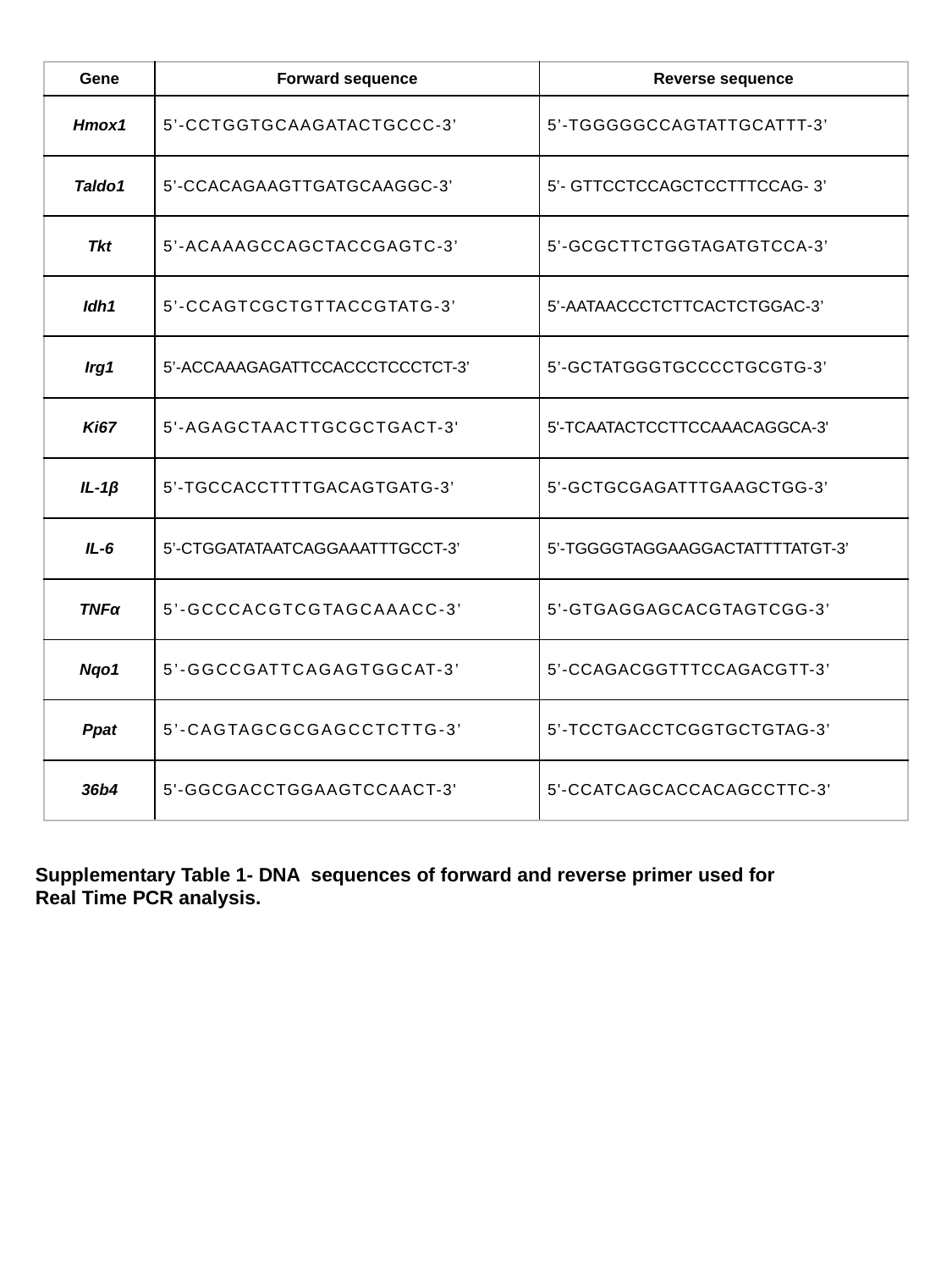

| Gene | Forward sequence | Reverse sequence |
| --- | --- | --- |
| Hmox1 | 5’-CCTGGTGCAAGATACTGCCC-3’ | 5’-TGGGGGCCAGTATTGCATTT-3’ |
| Taldo1 | 5’-CCACAGAAGTTGATGCAAGGC-3’ | 5’- GTTCCTCCAGCTCCTTTCCAG- 3’ |
| Tkt | 5’-ACAAAGCCAGCTACCGAGTC-3’ | 5’-GCGCTTCTGGTAGATGTCCA-3’ |
| Idh1 | 5’-CCAGTCGCTGTTACCGTATG-3’ | 5’-AATAACCCTCTTCACTCTGGAC-3’ |
| Irg1 | 5’-ACCAAAGAGATTCCACCCTCCCTCT-3’ | 5’-GCTATGGGTGCCCCTGCGTG-3’ |
| Ki67 | 5'-AGAGCTAACTTGCGCTGACT-3' | 5'-TCAATACTCCTTCCAAACAGGCA-3' |
| IL-1β | 5’-TGCCACCTTTTGACAGTGATG-3’ | 5’-GCTGCGAGATTTGAAGCTGG-3’ |
| IL-6 | 5’-CTGGATATAATCAGGAAATTTGCCT-3’ | 5’-TGGGGTAGGAAGGACTATTTTATGT-3’ |
| TNFα | 5’-GCCCACGTCGTAGCAAACC-3’ | 5’-GTGAGGAGCACGTAGTCGG-3’ |
| Nqo1 | 5’-GGCCGATTCAGAGTGGCAT-3’ | 5’-CCAGACGGTTTCCAGACGTT-3’ |
| Ppat | 5’-CAGTAGCGCGAGCCTCTTG-3’ | 5’-TCCTGACCTCGGTGCTGTAG-3’ |
| 36b4 | 5'-GGCGACCTGGAAGTCCAACT-3' | 5'-CCATCAGCACCACAGCCTTC-3' |
Supplementary Table 1- DNA sequences of forward and reverse primer used for Real Time PCR analysis.
